# Multi-Target Chemometric Modelling, Fragment Analysis and Virtual Screening with ERK Inhibitors as Potential Anticancer Agents

**DOI:** 10.3390/molecules24213909

**Published:** 2019-10-30

**Authors:** Amit Kumar Halder, Amal Kanta Giri, Maria Natália Dias Soeiro Cordeiro

**Affiliations:** LAQV@REQUIMTE/Department of Chemistry and Biochemistry, University of Porto, 4169-007 Porto, Portugal; amal.giri@fc.up.pt

**Keywords:** ERK inhibitors, QSAR, multi-target models, fragment analysis, virtual screening, molecular docking, molecular dynamics, binding free energy

## Abstract

Two isoforms of extracellular regulated kinase (ERK), namely ERK-1 and ERK-2, are associated with several cellular processes, the aberration of which leads to cancer. The ERK-1/2 inhibitors are thus considered as potential agents for cancer therapy. Multitarget quantitative structure–activity relationship (mt-QSAR) models based on the Box–Jenkins approach were developed with a dataset containing 6400 ERK inhibitors assayed under different experimental conditions. The first mt-QSAR linear model was built with linear discriminant analysis (LDA) and provided information regarding the structural requirements for better activity. This linear model was also utilised for a fragment analysis to estimate the contributions of ring fragments towards ERK inhibition. Then, the random forest (RF) technique was employed to produce highly predictive non-linear mt-QSAR models, which were used for screening the Asinex kinase library and identify the most potential virtual hits. The fragment analysis results justified the selection of the hits retrieved through such virtual screening. The latter were subsequently subjected to molecular docking and molecular dynamics simulations to understand their possible interactions with ERK enzymes. The present work, which utilises in-silico techniques such as multitarget chemometric modelling, fragment analysis, virtual screening, molecular docking and dynamics, may provide important guidelines to facilitate the discovery of novel ERK inhibitors.

## 1. Introduction

Mitogen-activated protein (MAP) kinases regulate a large variety of biological processes such as apoptosis, cell proliferation, motility, differentiation, mitosis, gene expression and immunity in response to growth factors and environmental stress [1]. The MAP kinase family consists of four major subfamilies of related proteins. Among these, the RAS/RAF/MEK/ERK signal transduction cascade (also referred as ERK pathway) was the first to be discovered, and it was found to play a crucial role in diverse cellular processes including cell proliferation, differentiation, migration and survival [2]. Aberration of the extracellular regulated kinase (ERK) pathway is associated with the prognosis of numerous human cancers including lung, kidney, ovary, colon and pancreas [3]. This pathway is therefore considered as an important therapeutic target for cancer treatment [4,5]. Since ERK exists at the end of this pathway to mediate various cellular processes, its inhibition should be the most effective to abort such pathway. More significantly, mutations of upstream regulators such as MEK or BRAF, which is frequently found to create resistance for MEK and BRAF inhibitors, hardly affect the activity of ERK inhibitors [6,7]. Therefore, ERK inhibitors are considered as promising agents for cancer therapy [5,8]. In mammals, two isoforms of ERK enzyme are found and these are ERK-1 and ERK-2, which share many but not all biological processes [8,9]. These two isoforms have >80% sequence identity and their ATP binding sites are also conserved [10]. Moreover, these isoforms are simultaneously activated in cellular systems and also demonstrate equivalent catalytic activity in vitro, indicating their functional redundancy [11,12]. Nevertheless, ERK-1 competes antagonistically with ERK-2 for MEK weakening its signalling. It is still unclear if these two isoforms have different substrates [8]. Despite many promises, only a few ERK inhibitors have entered different stages of clinical trials so far, thus demonstrating that the design and development of ERK inhibitors lagged far behind in comparison with the upstream inhibitors of the ERK pathway [8,13,14]. Currently ulixertinib (or BVD-523) is the most advanced ERK inhibitor that has depicted promising outcomes in clinical trials for the treatment of pancreatic cancer, acute myelogenous leukaemia and non-Hodgkin lymphoma [8,15,16]. Apart from ulixertinib, MK-8353, KO-947 and LTT-462 are in various stages of clinical development. Most of these agents simultaneously target both ERK-1 and ERK-2 and are referred as ERK-1/2 inhibitors [11,15,17]. 

Computational approaches are now considered as an integral part of the early stage of drug design and development [18,19,20]. Interestingly, only a few in silico investigations on the ERK inhibitors have been reported so far. These investigations were based on in silico methods such as 2D/3D-quantitative structure–activity relationships (2D/3D-QSAR), molecular docking, molecular dynamics simulations, etc [21,22,23]. However, these in silico investigations were performed with one of the isoforms of ERK and the number, as well as the structural diversity of the data points considered, have been also limited. The purpose of the present work is to develop multi-target chemometric models that may simultaneously predict the response parameters against both ERK isoform inhibitors (i.e., ERK-1 and ERK-2 inhibitors) under different experimental assay conditions [20,24,25,26,27]. Therefore, the scope of the proposed models will be not limited to only one isoform of ERK. By incorporating information from diverse inhibitors of both ERK-1 and ERK-2 assayed against multiple experimental conditions, these models attempt to provide meaningful explanations regarding the structural or physicochemical features required by potent ERK-1/-2 inhibitors. Furthermore, the current work also highlights important favourable and unfavourable fragments for the design of novel ERK inhibitors. Additionally, the QSAR model developed in the present work was utilised for virtual screening of a kinase library to obtain the most potential virtual hits as ERK-1/2 inhibitors. Finally, molecular docking calculations and molecular dynamics simulations were performed to confirm the results of the present drug design endeavour. 

## 2. Results and Discussions

### 2.1. Linear mt-QSAR Model

**Figure 1:**
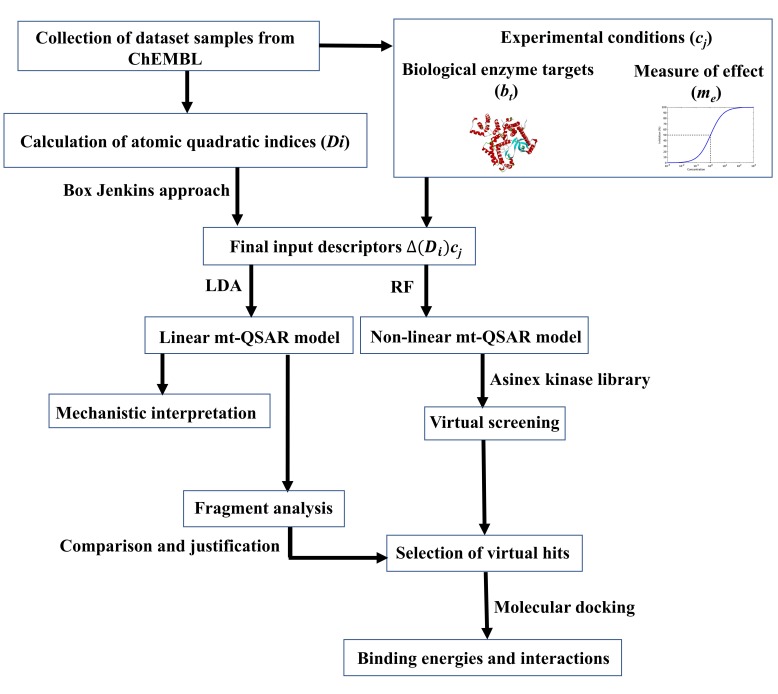
Flowchart showing the investigation performed in the current work.

A schematic representation of the workflow carried out here is presented in Figure 1. The dataset contained 6,400 ERK inhibitors, which were assayed against at least one of two ERK isoforms (i.e., ERK-1 and ERK-2). Moreover, the measure of effectiveness for these dataset compounds are either half-maximal inhibitory concentration (IC_50_) or binding affinity (Ki). The details of the dataset are provided in the Supporting Information (SI, file SM1.xlsx). The rationale and methodology of the Box–Jenkins based mt-QSAR modelling approach is described in the Materials and Methods section. Briefly, one of the most important factors considered for such multi-target models is their experimental elements, which are required to be decided before development of the model [27,28]. Two experimental elements, namely *bt* (biological target) and *me* (measure of effectiveness) are considered in the present analysis, depending on the nature of the current dataset. The *bt* element depends on the specific enzyme isoform (ERK-1 or ERK-2) against which the assay is performed whereas *me* is based on the type of measure of effect used for the response variable (IC_50_ or Ki). A combination of these two elements (i.e., *bt* and *me*) defines a specific experimental condition, which may be expressed as an ontology of the form *cj*→(*bt,me*). Therefore, the dataset contains some compounds which are assayed against more than one experimental element. In order to obtain categorical response variables *IAi*(*cj*), any dataset compound (*i*) with activity (IC_50_/K_i_) value ≤ 500 nM was assigned as active [*IAi*(*cj*) = +1] whereas the remaining considered as inactive [*IAi*(*cj*) = −1]. Notably, from the context of drug discovery, compounds exhibiting micromolar inhibitory potential are considered as ‘*hit*’ molecules [29]. The selected cut-off value, therefore, renders the developed models suitable for the selection of potent inhibitors. At the same time, this cut-off prevents excessive imbalance between active and inactive compounds. The software QUBILs-MAS v1.0 [30] was employed to calculate the molecular descriptors known as the atom-based quadratic indices, which have been earlier proved to be highly efficient for developing mt-QSAR models [27,31,32,33,34,35]. A detailed description of how these descriptors are calculated is provided in the Materials and Methods section. The linear and non-linear mt-QSAR models are developed separately with the help of genetic algorithm-linear discriminant analysis (GA-LDA) [36,37] and random forest (RF) methods, respectively, using the QSAR-Co tool [38]. QSAR-Co [39] is an open access Java-based tool developed by our group to facilitate the development of mt-QSAR models resorting to the Box–Jenkins approach. Before setting up these models, the whole dataset (*n* = 6400) was subjected to k-means cluster analysis (*k*-MCA) [40] to obtain a modelling set (*n* = 4481) and an external validation set (*n* = 1919). The setup of both linear and non-linear QSAR models are based solely on the modelling dataset, these being then validated with the external validation set compounds.

### 2.2. Linear Interpretable mt-QSAR-LDA Model

With the aim to develop an interpretable QSAR model, the GA-LDA technique was applied to the modelling dataset [38]. An interpretable QSAR model contains a limited number of molecular descriptors and these, therefore, may highlight the most significant structural and physicochemical factors important for the variation in response parameters [41,42]. The atom-based quadratic indices were employed to develop the linear models. For the model development, the modelling set was randomly divided into a sub-training set (*n* = 3585) and a test set (*n* = 896), using the QSAR-Co tool. The best linear mt-QSAR model found (a seven-variable equation) is shown below together with the statistical parameters of the GA-LDA.

*IAi*(*cj*) = 1.653 − 0.080 *D[Tnsq5(CH)_N2]m_e_* − 1.842 *D[Tnsq3(CH)_MN]b_t_* + 18.180 *D[Tssq11(CH)_MN]m_e_* − 0.027 *D[Tssq5(POL)_MX]m_e_* + 0.005 *D[Tnsq1(PSA)_GM]m_e_* + 0.003 *D[Tnsq13(VDW)_N2]b_t_* − 0.111 *D[Tssq2(HYD)_N1]m_e_* (1)

where *n*= 3585, λ = 0.397, Canonical *R* = 0.776, χ^2^ = 3302.20, *D*^2^ = 6.54, *p* < 10^−16^, and *F* (73,577) = 774.498.

The low Wilk’s lambda (λ) [41], the high values of the canonical *R* index, chi-square (χ^2^), and squared Mahalanobis distance (*D*^2^), overall indicate the goodness-of-fit and statistical significance of the developed model [42]. To go a step further and judge about the predictive power of this mt-QSAR classification model against both the sub-training and test sets, parameters such as the sensitivity, specificity, accuracy and the Matthews correlation coefficient (MCC) [42,43] were also examined (see Table 1).

**Figure 2:**
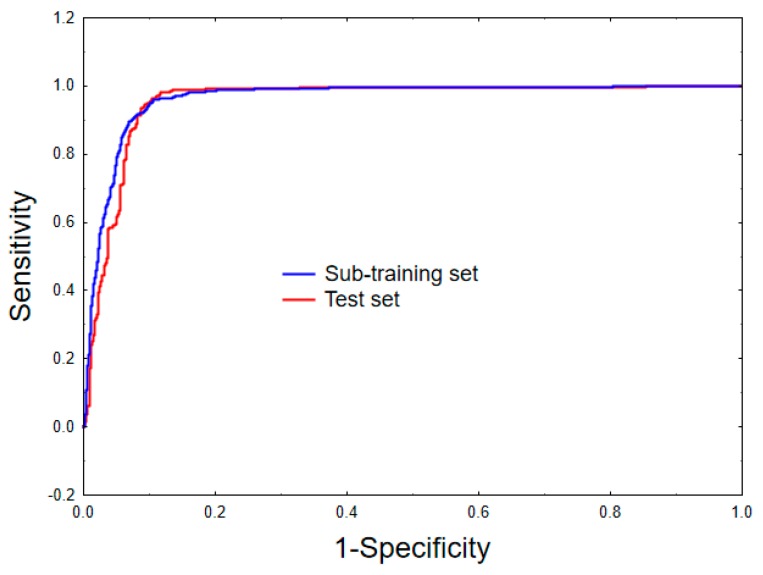
Receiver operating characteristic curves for the sub-training (ten-fold cross-validation) and the test sets.

As can be seen from Table 1, the model shows a satisfactory predictive ability as indicated by the values of the accuracy, MCC, along with the sensitivity, specificity and the *F*-measure obtained for both the sub-training and test sets [43,44,45]. This can also be judged by computing the area under the receiver operating characteristic (ROC) plots [46] shown in Figure 2. Indeed, the values attained for both the sub-training (ten-fold cross-validation) and test sets (i.e., 0.963 and 0.956, respectively), confirm once more the acceptable predictive power of the model. 

However, the reliability of every linear classification model does not merely depend on its predictability. Multi-collinearity, the applicability domain (AD) as well as the statistical robustness of the models should also be critically examined before judging the overall reliability of any mt-QSAR model [38,47]. To do so, the cross-correlation matrix of the independent variables included in the model was examined and it is presented in Table 2. As seen, the highest Pearson correlation (*r*) observed between two independent variables is 0.779. Therefore, it may be inferred that the model does not contain highly intercorrelated descriptors and it is non-redundant in nature. Next, the *Y*-randomization test [48] was performed in order to ensure that the model is not developed by chance. The average λ value of 100 randomised models was found to be 0.998, which is considerably higher than the original λ value obtained for the mt-QSAR model (i.e., 0.397), thus justifying its uniqueness. Finally, the AD of the developed model was estimated by a standardisation approach as suggested by Roy et al. [49]. A hundred and sixty-five compounds of the sub-training set and thirty-eight compounds of the test set were found outside the domain of applicability. Therefore, these compounds may be considered as possible outliers, but even so, no compound was removed in the current work on the basis of this AD analysis. 

After confirming that the developed linear mt-QSAR-LDA model fulfils the criteria for being a robust classification model, its true external predictability was determined by screening the external validation set (*n* = 1919), employing the QSAR-Co tool [38]. In so doing, it was found that 775 out of 791 active molecules and 1000 out of 1128 inactive compounds are correctly predicted by the model, leading therefore to an accuracy of 92.50%. This along with the MCC value attained (=0.854), implies also a satisfactory prediction ability of the model for the external validation set. Moreover, only sixty compounds of the external validation set were found to be outside the AD of the model. Altogether, these diverse statistics demonstrate the high internal quality as well as predictive power of the developed mt-QSAR-LDA model. All these results pertaining to this developed mt-QSAR-LDA model as well as its outliers are shown in SI (file SM1.xlsx). 

### 2.3. Interpretation of Molecular Descriptors

**Figure 3:**
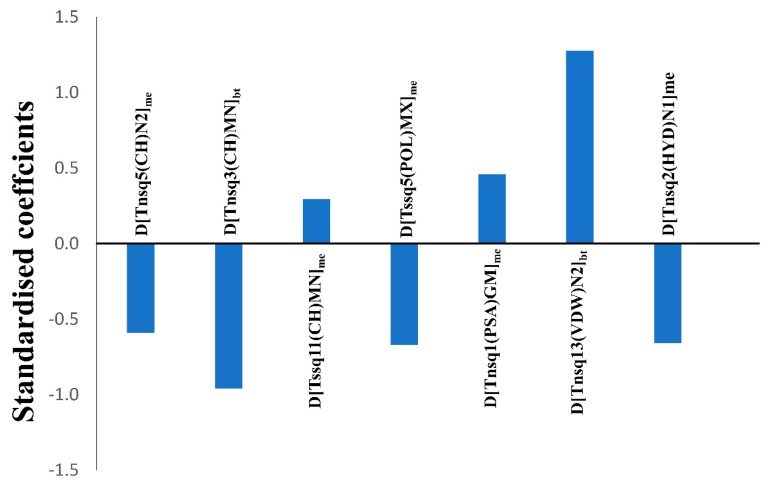
Standardised coefficients vs. variables in the mt-QSAR-LDA model.

Undoubtedly one of the major aspects of any QSAR linear model is its mechanistic interpretation [50], since its molecular descriptors may provide key insights about the structural requirements of a compound for having higher biological activity against one specific biological target under a particular experimental condition. Herein, we discuss the physicochemical/structural information of the molecular descriptors included in the linear mt-QSAR-LDA model with respect to their relative importance, by analysing the absolute values of their standardised coefficients. These standardised coefficients pertaining to the seven descriptors of the model are provided in Figure 3 whereas a description of their meaning is outlined in Table 3. The relative importance of such descriptors are as follows: *D[Tnsq13(VDW)N2]_bt_* > *D[Tnsq3(CH)MN]_bt_* > *D[Tssq5(POL)MX]_me_* > *D[Tnsq2(HYD)N1]_me_* > *D[Tnsq5(CH)N2]_me_* > *D[Tnsq1(PSA)GM]_me_* > *D[Tssq11(CH)MN]_me_*. 

It must be noted that the total atomic quadratic indices are calculated on the basis of the ‘topological distance’, which is simply the number of bonds (without considering bond multiplicity) present between any two atoms in a molecule [33,34]. Moreover, these descriptors of the model are modified from the originally calculated descriptors based on the Box–Jenkins approach (described below). Therefore, each descriptor is sensitive to two factors, namely: (a) Value of the core molecular descriptor, which is the calculated total atom-based quadratic index and (b) the experimental elements. Interestingly, the two most significant descriptors of the model are found to be sensitive to the experimental element *bt* (or biological target), whereas the remaining descriptors are dependent on the element *me* (or measure of effect). The most significant descriptor of the model is *D[Tnsq13(VDW)N2]_bt_*, which characterizes the increment of van der Waals volume by considering any two atoms separated at a topological distance equal to 13. A positive coefficient associated with this descriptor may indicate that by incrementing the steric volume of atoms linked with a topological distance of 13 favours higher activity. The second most important descriptor of the model is *D[Tnsq3(CH)MN]_bt_* and the negative coefficient of this descriptor suggests that by diminishing the charge between two atoms placed at a topological distance of 3 may favour higher activity. The third most important descriptor of the model is *D[Tssq5(POL)MX]_me_*, which also has a negative coefficient. It implies that by decreasing the polarizability between two atoms present at a topological distance of 5 may improve the ERK inhibition activity. Like charge and polarizability, hydrophobicity generally plays a crucial role in determining the biological profile of drug-like compounds. *D[Tnsq2(HYD)N1]_me_* is the only descriptor that is based on the atomic property hydrophobicity (Ghose–Crippen logP) [51] and this signifies that the hydrophobicity between two atoms separated at a topological distance of 2 should be decreased. Interestingly, three out of the seven descriptors of the model are based on the atomic property charge. One of these, named *D[Tnsq5(CH)N2]_me_* is the fifth most important independent variable of the model, which thus implies that the diminution of the charge between two atoms linked with an atomic distance of 5 favours higher activity. The sixth most important descriptor of the model *D[Tnsq1(PSA)GM]_me_* is based on the physicochemical property polar surface area. Notably a positive coefficient was found for this descriptor in the model, and therefore that indicates that an increment of the polar surface area linked with a topological distance of 1 improves the activity. The least important descriptor of the model is *D[Tssq11(CH)MN]_me_*, which is positively correlated with the biological activity. This thus indicates that an increase of the charge between two atoms linked with a topological distance of 11 should be higher in order to obtain higher inhibitory activity against ERK enzymes. Overall, it is observed that when atoms are present at shorter distances (topological distances 1–5), their overall charge, polarizability and hydrophobicity should be lower, but their overall polar surface area should be higher. When the atoms are separated by longer distances (topological distances 6–15), their overall steric volume, as well as charge, should be increased. 

### 2.4. Quantitative Contributions of the Molecular Fragments

**Figure 4:**
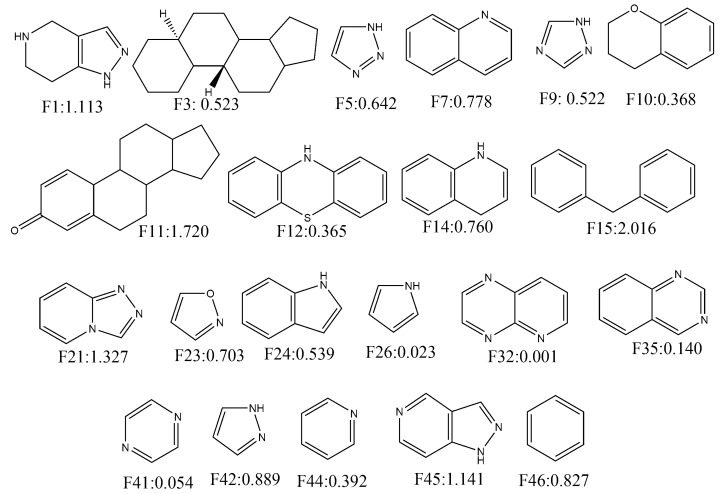
Fragments depicting positive contribution for extracellular regulated kinase (ERK)-1/2 inhibition with their average confidence scores.

In this work, we utilised the built mt-QSAR-LDA model to understand the contributions of different molecular fragments for higher/lower activity towards ERK inhibition. For this, the whole dataset (*n* = 6400) was used to collect the Bemis–Murcko scaffolds [52] by using the OCHEM web server [53]. Forty-six single ring fragments were then identified from the current dataset on the basis of their frequencies (present in more than 15 compounds) in the dataset compounds. The molecular descriptors of Equation (1) were then calculated for these fragments. For each fragment four different types of experimental conditions were considered, and these are c1 (bt: ERK-1, me: IC_50_), c2 (bt: ERK-1, me: Ki), c3 (bt: ERK-2, me: IC_50_) and c4 (bt: ERK-2, me: Ki). Notably, all these four conditions are found in the dataset on which the LDA model was developed. A total of 184 (= 46 × 4) scores were obtained by putting the calculated variables into Equation (1). These scores are however non-standardised, and the following standardisation procedure was employed to obtain standardised scores. The average and standard deviation of the non-standardised scores were calculated and each non-standardised score is subtracted from the average score and these subtracted values were subsequently divided by the standard deviation. These standardised scores (or confidence scores) represent the quantitative contributions of the fragments for the inhibitory potentials of these fragments and various experimental conditions [25,26,27,35,42,54]. The confidence scores obtained for four different assay conditions were then averaged to obtained average confidence scores (ACS). Twenty-one fragments showing positive average confidence scores are depicted in Figure 4. Fragments such as **F15, F11, F21, F45, F1, F42, F46, F7, F14** and **F23** showed highly positive confidence scores (>0.70). Therefore, these fragments may be considered for the design of novel ERK-1/2 inhibitors. Interestingly, most of the fragments showing positive contributions are either bulky in nature or have aromaticity. Moreover, steroidal structures (**F3** and **F11**) showed positive contributions towards higher activity. Notably in mt-QSAR-LDA model, descriptor containing van der Waals volume (i.e., *D[Tnsq13(VDW)N2]_bt_*) was found to have the most significant positive contribution to the increase of biological activity. Therefore, it may be inferred that the mt-QSAR-LDA modelling results are consistent with the ones coming from the fragment analysis since both suggest the high significance of steric groups for better activity.

**Figure 5:**
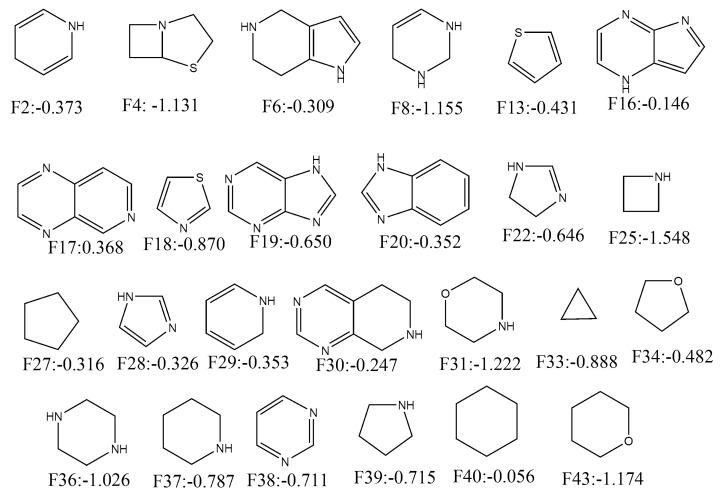
Fragments depicting positive contribution for ERK-1/2 inhibition with their average confidence scores.

Similarly, 26 fragments with negative ACS are presented in Figure 5. As can be deduced, fragments such as **F38, F39, F37, F18, F33, F36, F4, F8, F43, F31** and **F25** have significantly high negative contributions (<−0.70). Interestingly, all alicyclic fragments (i.e., **F4, F25, F27, F31, F33, F34, F36, F37, F39, F40** and **F43**) demonstrated negative contributions in our analysis. These fragments mainly interact through hydrophobic interactions (though for some fragments hydrogen bonding interactions may take place). It matches the former interpretation of our mt-QSAR-LDA model where hydrophobicity (obtained from specific topological distances) was found to be negatively correlated with the enzyme inhibitory potential. As far as the aromatic rings are concerned, benzene as a single fragment (i.e., **F15** and **F46**) or part of polycyclic rings (**F7, F10, F12, F14, F24** and **F35**) showed positive ACS. However, **F20** is an exception. Nevertheless, for other heterocyclic aromatic rings no such definite conclusion may be made as some of these fragments showed positive contributions whereas other fragments depicted negative ones. This reflects the complex relationship between the enzyme inhibitory activity and the molecules’ charge distribution just as observed in our mt-QSAR-LDA model. It is, however, worth mentioning here that quantitative contributions of these all fragments are relative. The scores may be altered if these are connected to other fragments. In such situation, the derived model may also be used for the calculation of multiple ring fragments to understand their contributions. The results pertaining to the current fragment analysis are provided in SI (file SM2.xlsx).

### 2.5. Non-Linear mt-QSAR-RF Model

Following on, we applied the random forest (RF) technique [55,56] to develop a non-linear predictive model, using the QSAR-Co tool [38]. It is often observed that when non-linear machine learning techniques are employed with all calculated descriptors, these generate highly predictive QSAR models, of course at the expense of lacking overall interpretability [41,42,57,58]. RF classification models are developed by generating a forest of decision trees using the modelling set. RF models are considered as robust as well as highly predictive. However one the major advantages of RF over other non-linear machine learning methods is that, to a large extent, it restricts the model from overfitting [58,59]. Here, the modelling set (*n* = 4481) used for developing the linear model was also used for generating the mt-QSAR-RF model, and the statistical results of this model are depicted in Table 4. 

As seen in Table 1 as well as in Table 4, the statistical quality of the mt-QSAR-RF is considerably higher than that of the mt-QSAR-LDA model. The RF model could correctly predict 1239 out of 1306 active compounds as well as 2209 out of 2279 inactive compounds of the sub-training set, after ten-fold cross-validation. At the same time, 304 out of 316 active compounds and 559 out of 580 inactive compounds of the test set are correctly predicted by this model. Therefore, the RF model afforded significantly high accuracy values of 96.18% and 96.32% for the sub-training and the test sets, respectively. The MCC values obtained for the sub-training and test sets are 0.918 and 0.920, respectively. Additionally, the high area values of the ROC plots calculated for both the sub-training and test sets (0.990 and 0.987, respectively) clearly indicate that a strong correlation exists between the observed and predicted categorical values. 

In order to understand whether the external predictability of the RF model is as good as its internal predictability, the model was used to screen the external validation set (*n* = 1919). The model successfully predicted 762 out of 791 active and 1093 out of 1128 inactive compounds achieving values for the sensitivity, specificity, accuracy and MCC of 96.33%, 96.90%, 96.70% and 0.931, respectively. These statistical values strongly indicate the high discriminatory power of this mt-QSAR-RF model.

### 2.6. Virtual Screening with Kinase Database

**Figure 6:**
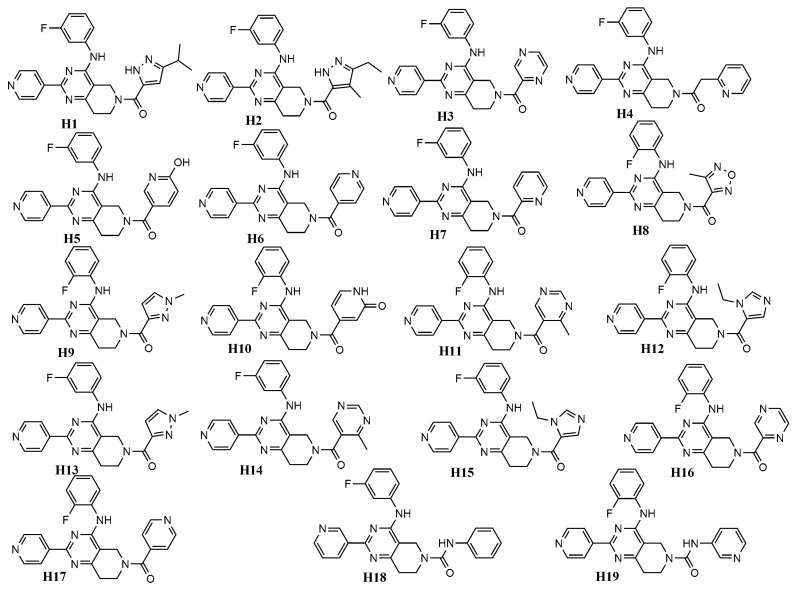
Chemical structures of virtual hits (**H1**–**H19**) for ERK-1/2 inhibition.

Considering its high predictability, we applied the developed mt-QSAR-RF model to perform a virtual screening with a focused library named Asinex Kinase Library [60], which contains 6538 compounds. The structures of all these database compounds are depicted in the Supporting Information (file SM3.xlsx). Similar to the fragment analysis, each of these database compounds was assigned with four different types of experimental conditions, which are c1 (bt: ERK-1, me: IC_50_), c2 (bt: ERK-1, me: Ki), c3 (bt: ERK-2, me: IC_50_) and c4 (bt: ERK-2, me: Ki). After screening with the RF model, only 1255 out of these 26,152 (=6538 × 4) cases were found to be predicted as active (i.e., *IAi*(*cj*) = +1). After scrutinising these positive/active cases, we observed that only 19 compounds demonstrated positive activity for all these four experimental conditions (i.e., c1–c4) (These results are provided in the Supporting Information, file SM4.xlsx.). These 19 compounds (named as **H1**–**H19**) are considered as the most potent virtual hits (for ERK inhibition) and their structures are shown in Figure 6. 

Figure 6 clearly indicates that all these top hit molecules are structurally similar to each other. Each molecule contains four ring fragments while three-ring fragments named 5,6,7,8-tetrahydropyrido [3,4-d]-pyrimidine, pyridine and benzene are common in all these structures. Interestingly, these common fragments were previously analysed in Section 2.4. Therefore, all these three fragments were also observed multiple times (>15 dataset compounds) in the dataset used for developing the QSAR models. Both pyridine (**F44**, ACS:0.392) and benzene (**F46**, ACS:0.827) show positive ACS but 5,6,7,8-tetrahydropyrido[3,4-d]-pyrimidine (**F30**, ACS:−0.247) has a slight negative score. It is also interesting to find that many of the previously analysed fragments are found in the hit molecules and these are **F42** (ACS:0.889), **F41** (ASC:0.054), **F38** (ASC:−0.711), **F28** (ASC:−0.326) and **F29** (ASC:−0.353). **H8** is the only hit molecule, one fragment of which is not analysed in fragment analysis (see Section 2.4). Furthermore, adding all ACS values of these fragments we get an overall positive score for all other 18 virtual hits (i.e., **H1**–**H7, H9**–**H19**).

### 2.7. Molecular Docking Analysis

To understand how these 19 hit molecules may interact with the ERK enzymes, we performed molecular docking calculations. The X-ray crystal structures of ERK-1 (PDB ID: 4QTB [61] and ERK-2 (PDB ID: 4QTA [61]) were used separately for the docking of these 19 hit molecules. These two protein structures were reported with a very low resolution of 1.40 and 1.45 Å, respectively. Moreover, both these protein structures are bound with the ligand SCH772984, which apart from being a selective inhibitor of ERK1/2, also has characteristics of both type I and type II kinase inhibitors [61,62]. SCH772984 utilises three different binding pockets (i.e., adenine mimetic pocket, ribose/phosphate pocket and p-loop pocket) for interacting with ERK-1/2 enzymes [61]. In order to understand the binding cavity, we performed blind docking calculations with the virtual hits using the Autodock Vina software [63]. In this blind docking, the whole structures of the enzyme crystal structures were taken into consideration for docking of the hit molecules. Interestingly, it was observed that all these hit molecules preferably bind at the binding cavity of SCH772984. The blind docking was followed by a rigid docking experiment performed with Autodock 4.2 [64] to estimate the binding energies as well as to understand possible interactions of these hits with the enzymes. The binding energy of the virtual hits (**H1**–**H19**) obtained in the rigid docking is presented in Table 5.

**Figure 7:**
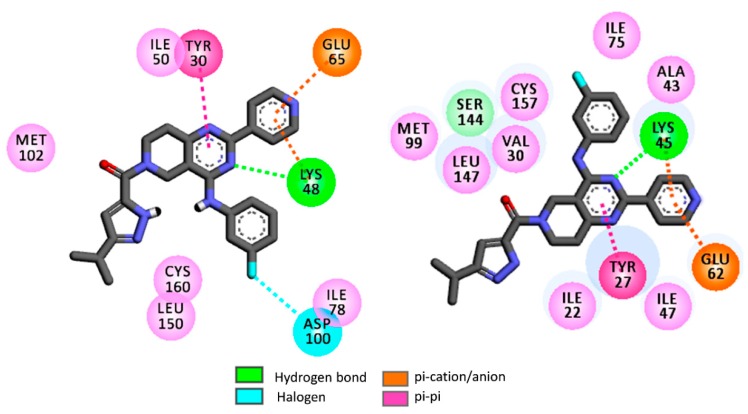
2D rigid docking interaction diagrams of H1 with ERK-1 or 4QTB (**left**) and ERK-2 or 4QTA (**right**).

In rigid docking, all these hit molecules (**H1**–**H19**) showed similar binding energies against the ERK enzyme isoforms. Moreover, these binding energies are comparable to binding energies of the reference compound ulixertinib. The 2D docking interactions diagrams of **H1** with 4QTB (ERK-1) and 4QTA (ERK-2) are presented in Figure 7.

Notice that the 5,6,7,8-tetrahydropyrido[3,4-d]-pyrimidine residue of **H1** displays a hydrogen bond interaction with the catalytic Lys48 residue of ERK-1, as well as in a similar fashion with the catalytic Lys45 residue of ERK-2. These catalytic lysine residues also foster π-cation interactions with the pyridine moiety of **H1**. Apart from this, Tyr27 of ERK-2 and Tyr30 of ERK-1 were found to be important residues as these establish strong π-π interactions with the 5,6,7,8-tetrahydropyrido[3,4-d]-pyrimidine moiety of **H1**. Apart from these, this rigid docking confirms that a number of hydrophobic interactions are possible between **H1** and the binding site amino acids of ERK-1/2.

It is well known that kinases may adopt multiple conformations depending on the ligand structures. ERK enzymes are not an exception since, for instance, it has been reported that the isoform ERK-2 may adopt multiple conformations depending on the nature of the ligands [65]. In both 4QTA and 4QTB, Tyr27/Tyr30 are present as ‘in’ conformations. As an example, Tyr27 of 4QTA tucks under the glycine-rich loop. The π-π stacking interactions that are obtained between tyrosine residues (i.e., Tyr27 and Tyr30) and 5,6,7,8-tetrahydropyrido[3,4-d]-pyrimidine residues of **H1** may thus appear because of ‘in’ conformations of these tyrosine residues [61,65]. However, it was observed that Tyr27 ‘out’ conformation also exists in ERK-2 where Tyr27 on the glycine-rich loop is engaged in π-π stacking interactions with Tyr55 of the C-α helix [65]. In addition, the polar side chains of the Lys48/Lys45 residues in ERK-1/2 are also highly flexible in nature. Therefore, in order to further elaborate the interaction patterns of these virtual hits, flexible docking was performed with the help of Autodock software [64]. In the flexible docking of ERK-1, flexibility was imparted to the side chains of Lys48 and Tyr30 residues. Similarly, the Lys45 and Tyr27 were rendered flexible in the ERK-2 crystal structure. The binding energies obtained in the flexible docking are presented in Table 5.

**Figure 8:**
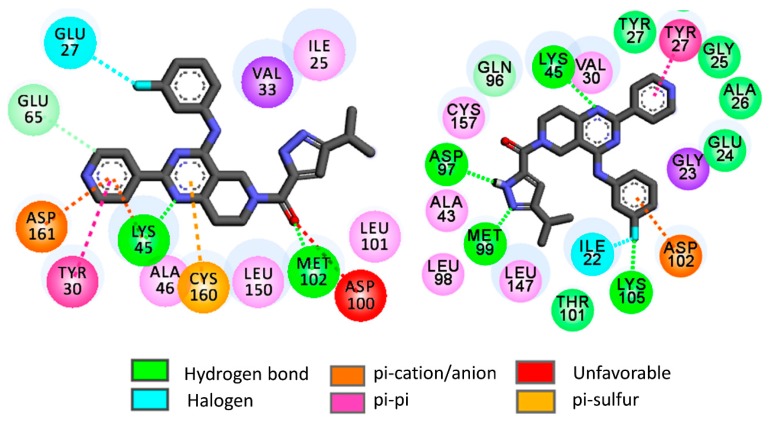
2D flexible docking interaction diagrams of **H1** with ERK-1 or 4QTB (**left**) and ERK-2 or 4QTA (**right**).

As compared to rigid docking, flexible docking yielded slightly higher binding energies for most of the hits. Moreover, the binding energies of these hits are close to each other as well as with the reference compounds ulixertinib. However, the ligand-receptor interactions obtained in flexible docking varied considerably from the interactions obtained in rigid docking. As an example, the interaction obtained for **H1** in the flexible docking is presented in Figure 8.

In ERK-2, **H1** forms hydrogen bond interactions with binding site amino acids such as Met99, Asp97, Lys45 and Lys105. The pyridine moiety of **H1** establishes strong π-π interactions with Tyr27 whereas π-anion interactions are set up between the fluorobenzene moiety of **H1** and Asp102. On the other hand, the **H1** docked pose in ERK-1 depicts hydrogen bond interactions with Met102 and Lys71. At the same time, the pyridine moiety of **H1** undertakes π-π, π-cation as well as π-anion interactions with Tyr30, Lys45 and Asp161, respectively. It is worth mentioning here that Tyr30 of ERK-1 and Try27 of ERK-2 are found as ‘out’ conformations in the flexible docking of these virtual hits. Therefore, it may be inferred that the binding of these virtual hits may prefer the ‘out’ conformations of these tyrosine residues. However, in the flexible docking of the reference compound ulixertinib, these tyrosine residues are found as ‘out’ conformations. It is worth mentioning that this observation complies with experimental results where ulixertinib favoured the ‘out’ conformation of Try27 in ERK-2 [65].

The fluorobenzene residue establishes halogen mediated interactions with Glu27 residue of ERK-1. Overall, the flexible docking indicates that the binding pattern may alter considerably when the flexibilities of binding site amino acids are taken into consideration. These results thus encouraged us to perform a molecular dynamics (MD) simulation with selected docked complexes to understand the dynamic behaviour of the virtual hits within the ERK enzymes.

### 2.8. Molecular Dynamics Analysis

We performed 10 ns molecular dynamics (MD) simulations with ERK2-H1 and ERK1-H1 complexes obtained from the rigid docking experiments. As references, the docked poses of ulixertinib in ERK-1 (ERK1-ULX) and ERK-2 (ERK1-ULX) obtained were also subjected to MD simulations. The root-mean-square-deviation (RMSD) of the backbone atoms of the receptor-ligand complexes as well as the root-mean-square-fluctuation (RMSF) plots of these protein structures are presented in SI (Figures S1–S3). These diagrams confirm that all these complexes achieved sufficient dynamic stabilities during the simulation. Similarly, the radius of gyration’ plots (see Figure S4 of SI) also indicates enough compactness of these macromolecule complexes. In addition, the stability of the ligands is also confirmed from the RMSD values of the ligands, presented in SI (Figure S5). The binding free energies of the ligands obtained through MM-GBSA analyses are given in Table 6.

**Figure 9:**
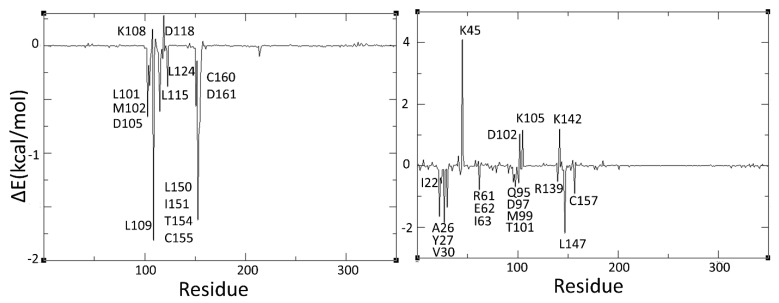
Per-residue decomposition profiles of ERK1-H1 (**left**) and ERK2-H1 (**right**) complexes.

Note that the ERK-2 bound complexes of **H1** and the reference compound (i.e., ulixertinib) have higher binding energies (i.e., −33.46 and −27.38 kcal/mol, respectively) as compared to their respective ERK-1 complexes (−23.28 and −21.38 kcal/mol, respectively). It is important to note here that ulixertinib, which is an ATP competitive kinase selective inhibitor, depicts 7.5 times higher inhibitory potential against ERK-2 (i.e., 40 pM) compared to ERK-1 (300 pM) [66]. Therefore, the binding free energies obtained for ulixertinib in our MM-GBSA analysis are consistent with the experimental results. Interestingly, similar binding free energy results are obtained for the **H1** complexes of ERK isoforms. That is, the binding free energy analysis shows that **H1**, as well as the other virtual hits identified in this work (i.e., H2–H19), may act as potent inhibitors of ERK-1/2. An attempt has been made to identify major residues involved in the ligand–protein interactions through per-residue energy decomposition analysis. The per-residue energy decomposition plots of ERK1-H1 and ERK2-H2 are provided in Figure 9.

Such analyses reveal that the interactions of **H1** with ERK-2 is favoured by residues such as Ile22, Ala26, Tyr27, Val30, Arg61, Glu62, Ile63, Gln95, Asp97, Met99, Trp101, Arg139, Leu147 and Cys157, whereas Lys45, Asp102, Lys105 and Lys142 are the main residues that disfavoured its binding. Significantly, most of these interactions are predicted by molecular docking calculations (Figure 7 and Figure 8). Complying with the binding energy results, fewer interactions are obtained when **H1** is complexed with ERK-1. Note that residues such as Leu101, Met102, Asp105, Leu109, Leu115, Leu124, Leu150, Ile151, Trp154, Cys155, Cys160 and Asp161 favoured binding of **H1** in ERK-1. At the same time, Lys108 and Asp118 disfavoured its binding. These energy decomposition results of ERK1-H1 are more consistent with the results coming from the flexible docking rather than the ones from the rigid docking.

For comparison and validation, the per-residue decomposition profile of ERK2-ULX complex was also analysed and the decomposition analysis plot is presented in SI (Figure S6). As can be seen, this plot resembles the per-residue decomposition plot of ERK2-H1 complex (Figure 9). More importantly, most of the residues which were found to be important in the binding of ulixertinib have been earlier reported in the ulixertinib-bound ERK-2 crystal structure (PDB:6GDQ) [65].

### 2.9. Assessment of Drug-Likeness

To estimate the drug-likeness of the proposed virtual hits, molecular descriptors like molecular weight (*MW*), number of hydrogen bond donor (*nHDon*), number of hydrogen bond acceptor (*nHAcc*) and lipophilicity (*ALOGP*) were calculated for these hits with the help of the software Dragon [67]. As seen in Table 7, the values of these physicochemical properties justify that all these hit molecules comply with the Lipinski’s rule of five [68], which states that in order to exhibit good oral bioavailability, a compound should have a molecular weight (*MW*) less than 500 Da, no more than 5 hydrogen bond donors (*nHDon*), no more than 10 hydrogen bond acceptors (*nHAcc*) and a logarithm of the octanol–water partition coefficient (*ALOGP*) less than 5.

## 3. Materials and Methods

### 3.1. Dataset Curation and Descriptor Calculation

After collecting the reported ERK-1/2 inhibitors from CHEMBL (https://www.ebi.ac.uk/chembl/), the dataset was curated by removing duplicate data-points. The SMILES formats of the molecules obtained from the CHEMBL were converted into SDF formats by the MarvinView v18.18.0 software (https://docs.chemaxon.com/display/docs/MarvinView). The atom-based quadratic indices were calculated by the software QUBIL-MAS v1.0, a freely available webserver for QSAR descriptor calculation. These descriptors have been used in different fields of research associated with drug discovery [25,26,27]. At present, the quadratic indices are calculated according to the following formalism:

(2) $$Lqkx=\overset{n}{\underset{j=1}{\sum }}{}^{k}a_{ij}x_{i}x_{j}$$

where *Lqkx* is the quadratic index of order *k* which considers the atom *i* and its chemical environment with respect to its neighbour atoms at the topological distance *k*. The term *^k^a_ij_* represents the adjacency between the atoms of the molecule. The *x* term characterises the physicochemical property considered for calculation of the descriptors. In this work, eight properties were considered, namely: hydrophobicity or Ghosh Crippen logP (HYD), charge (CHR), electronegativity (E), mass (M), polarizability (P), polar surface area (PSA), refractivity (R) and van der Waals volume (VDW). As it can be seen from Equation (2), the quadratic indices are calculated for each atom of the molecules, and a few different mathematical techniques may be adopted for the calculation of the total quadratic indices (*TsqxMT*), these being as follows:

(3) $$\text{TsqkN1}\;=\;\sum_{i=1}^{n}Lqkx,$$

(4) $$\text{TsqkN2}\;=\;\sum_{i=1}^{n}\sqrt{{\left(Lqkx\right)}^{2}},$$

(5) $$\text{TsqkGM}=\sqrt[{n}]{\prod_{i=1}^{n}(Lqkx)},$$

*TsqkRA* = *Lqkx_max_* − *Lqkx_min_*. (6)

In these equations, *Tsqkx* may either refer to the total non-stochastic quadratic index (represented as *Tnsqkx*) calculated from the non-stochastic adjacency matrix. Similar strategies are adopted for stochastic adjacency matrix-based descriptors, which are represented as *Tssqkx*. In Equation (3) and Equation (4), *N1* an *N2* refer to the Manhattan distance and the Euclidean distances, respectively. Equation (5) represents geometric mean (*GM*) based calculations of the total quadratic indices whereas Equation (6) depicts range-based (*RA*) ones. The maximum (*MX*) and the minimum (*MN*) values are used for the calculation of the later and these maximum and minimum values may also be used for the calculation of the total quadratic indices. Here, the total quadratic indices were calculated based on *N1, N2, GM, RA, MX* and *MN* techniques.

### 3.2. Box–Jenkins Approach

Although the calculated total quadratic indices characterise the chemical structures of the compounds, these descriptors fail to incorporate the influence of the multiple experimental conditions on chemical structure. This problem may be sorted out by the Box–Jenkins moving average approach, which has been largely discussed previously in detail [27,28,35,38,47]. Briefly, in Box–Jenkins based mt-QSAR modelling, the calculated descriptors (or *D_i_*) are modified to obtain deviation descriptors (∆(*D_i_*)*cj*), which represent the structural attributes of the compounds as well as the experimental conditions *c_j_*. Therefore, ∆(*D_i_*)*c_j_* allows estimating to what extent a compound may structurally deviate from a set of compounds assigned as active and tested against the same experimental condition [34,38,54,69]. In this work we used our recently launched QSAR-Co tool [38,39] to automatically calculate the ∆(*D_i_*)*c_j_* descriptors with the input descriptors *D_i_*.

### 3.3. Model Development and Validation

The QSAR-Co tool was also used for developing the mt-QSAR models by employing both GA-LDA and RF methods [38]. Before setting up both these models, the dataset was divided into a modelling set and an external validation set by the k-means cluster analysis (k-MCA) technique [40] with the help of the STATISTICA software [70] to ensure that both these sets have similar chemobiological spaces [47]. For k-MCA, the calculated total quadratic indices (D_i_) and the IAi(cj) values were used for generating 10 clusters based on the Euclidian distances from 500 iterations. From each cluster, external validation set samples were randomly collected to build an external validation set of 1919 data samples. It is worth mentioning here that the mt-QSAR models were developed only with the remaining 4481 samples used as the modelling dataset. Once the models are developed with the modified descriptors, these were then used to screen the external validation set in order to estimate their true predictivity [28]. The best predictive model was selected based on the predictivity obtained for the external validation set. For setting up the models, however, the modelling dataset was further randomly divided into a sub-training (80% of the training data) and a test set (20% of the training data) with the help of the QSAR-Co tool [38].

The parameter settings used for the GA-LDA technique in QSAR-Co were: (a) total number of iteration/generation: 100, (b) equation length: 10 (fixed), (c) mutation probability: 0.3, (d) initial number of equation generated: 100, (e) number of equation selected in each generation: 30. Similarly, important parameter settings of QSAR-Co for RF modelling were: (a) each bag size: 100, (b) maximum depth: 0 (unlimited), (c) number of randomly chosen features: 0 [i.e., *n* = int(log2(#Predictors) + 1)], (d) number of iterations: 100. It should be noted that changes in these parameter settings failed to improve the predictivity of the modelling dataset to a considerable extent. During development of both GA-LDA and RF models, data pre-treatment was carried out where descriptors containing intercorrelation (r^2^) of more than 0.85 and variations less than 0.001 were removed. Statistical indices such as the Wilks’ lambda (λ), chi-squared (χ^2^), the square of Mahalanobis distance (D^2^), Fisher’s statistic index (F) and the corresponding p-value (p) were calculated by the STATISTICA software [49] to estimate the goodness-of-fit of the LDA model [43]. The goodness of prediction for the sub-training, test and external validation sets was evaluated by computing the following statistical measures: sensitivity (correct classification of the active cases), specificity (correct classification of inactive cases), accuracy (overall correct classification), F-measure and Matthews correlation coefficient (MCC) [43,44]. Moreover, a Y-randomization test was carried out on the sub-training set by QSAR-Co to check the uniqueness of the statistical model [47,48]. Therefore, the values of the dependent variable were randomly scrambled 100 times, and the Wilk’s lambda (λ) of the original model was then compared with the average Wilk’s lambda (λ_rand_) of the randomized models. For determining the applicability domain, the standardisation approach [49] was employed with the help of the QSAR-Co tool [38].

### 3.4. Molecular Docking Analysis

The X-ray crystal structures of ERK-1 (PDB:4QTB) and ERK-2 (PDB:4QTA) were obtained from the Protein Data Bank [71,72]. The non-terminal missing amino acid residues of 4QTA were filled with the help of Modeller package [73] in Chimera software (version 1.12, University of California, San Francisco, CA, USA) [74]. The protonation states of amino acid residues of all these protein structures were fixed at pH = 7.0 with the help of PropKa server [75]. The blind docking calculations of the virtual hits were performed by the Autodock Vina tool (version 1.1.2., The Scripps Research Institure, La Jolla, CA, USA) [63]. The protein structures were prepared by removing all water molecules and ligands. For both protein and ligands, the partial atomic charges were assigned using the Gasteiger–Marsili method [76]. A grid box was centred on the macromolecules with 120 Å × 120 Å × 120 Å dimensions. The blind docking calculation was performed with an exhaustiveness value of 45. The rigid and flexible docking experiments are performed using Autodock 4.2 (The Scripps Research Institure, La Jolla, CA, USA) [64]. A grid map with 60 Å × 60 Å × 60 Å with a grid-point spacing of 0.375 Å were defined from the blind docked poses. A genetic algorithm-based conformational search was performed in both rigid and flexible dockings. However, in the rigid docking, the maximum number of evaluations was set to 2,500,000, and this was increased to 25,000,000 for the flexible docking. Other important genetic algorithm parameters used for docking are as follows: (a) number of runs: 10, (b) population size: 150, (c) maximum number of generations: 27,000, (d) rate of gene mutation: 0.02, (e) rate of cross-over: 0.8 (method twopt). Default docking parameter settings found in Autodock 4.2 were used for both the rigid and flexible docking. Analysis of the 2D ligand protein interactions was conducted using the Discovery Studio Visualizer 2017 R2 [77].

### 3.5. Molecular Dynamics Simulation

We have performed molecular dynamics (MD) simulations for four different protein-ligand complexes, i.e., ERK1-H1, ERK1-ULX, ERK2-H1 and ERK2-ULX. The initial structures of the complexes were obtained from the results of the molecular docking simulations and placed each of them at the centre of a three-dimensional periodic box of size 9.3 nm × 9.3 nm × 9.3 nm. Thereafter, the boxes were filled with 25,000 water molecules, and few Na^+^ ions were added to naturalize the charge of the complexes.

All the MD simulations were performed using the GROMACS-5.1.4 software package (Uppasala University, Stockhome, Sweden) [78,79]. The GROMOS96-54a7 force filed parameters were used for both proteins and ions, whereas the PRODRG server [80,81] was used to generate force field parameters for the ligands. The SPC/E water model [82] was used for describing the water–water interactions, and geometric combination rules were used to calculate cross interactions between unlike atoms considering the recent work by Giri et al. [83,84,85]. Initially, the systems were optimised for 5000 steps with a time constant of 1 fs using the steepest descent algorithm. Thereafter, the systems were equilibrated in two steps: (i) first, 100 ps in the *NVT* ensemble were performed to reach a stabilised system’s temperature at 300 K; (ii) then, the systems were equilibrated for 100 ps in *NpT* ensemble to stagnate the pressure of the system at 1 bar. After completion of the equilibration steps, the systems were simulated for 10 ns in *NpT* ensemble at 300 K to collect data for analysis. During this final simulation, the leap-frog algorithm [86] with a time step of 2 fs was used to integrate the equations of motion. The pressure of the system was controlled using the Parrinello–Rahman method [87] with a time constant of 2 ps. We used the v-rescale coupling algorithm [88] with a time constant of 0.1 ps to keep the system’s temperature at the intended value of 300 K. The short-range Lennard–Jones potential and Coulombic interactions were cut off at 1 nm, and the long-range Coulombic interactions were computed by the Particle Mesh Ewald (PME) method [89,90].

Binding free energy (∆*G_bind_*) of the ligands was evaluated using the Molecular Mechanics Poisson Boltzmann Surface Area (g_mmpbsa) method [91] implemented in GROMACS 5.1.4. The binding free energy can be expressed as follows:

(7) $$\Delta G_{bind}=G_{complex}-\left(G_{protein}+G_{ligand}\right)$$

where *G_complex_* is the total free energy of the complex, and *G_protein_* and *G_ligand_* are the total free energy of the separately solvated protein and ligand, respectively. The free energy (*G_x_*) of the individual species, *x*, can be represented as,

(8) $$G_{x}=<E_{MM}>-TS+<G_{solvation}>$$

where $E_{MM}=E_{bonded}+E_{non-bonded}=E_{bonded}+(E_{vdw}+E_{elec})$. The <*E_MM_*> is the average molecular mechanics’ potential energy in vacuum, which is the sum of the bonded, van der Waals and electrostatic interaction potentials. The solvation free energy, *G_solvation_*, can be expressed as the sum of the electrostatic solvation free energy (*G_polar_*) and non-electrostatic solvation free energy (*G_non-polar_*). *G_polar_* is estimated solving Poisson–Boltzmann equation, whereas *G_non-polar_* is calculated from the solvent-accessible surface area (SASA) using the following equation,

(9) $$G_{non-polar}=\gamma SASA+b$$

where *γ* and *b* are the empirical constants.

## 4. Conclusions

Machine learning techniques may effectively extract crucial information from large complex diverse datasets and are now regularly employed for the design of new therapeutic agents [19]. Taking advantage of the ever-expanding chemical libraries and the latest advances in machine learning techniques, potential drug-like candidates may be identified in an efficient and cost-effective way. Machine learning-based multi-target QSAR modelling, which truly integrates the chemical library data for simultaneous prediction of response variables under various experimental assay conditions, have been successfully employed in the last few years to develop chemometric models against various biological targets [27,28,33,34,35,38,47,50]. In this work, we developed linear and non-linear mt-QSAR models with a large dataset containing ERK-1 and ERK-2 inhibitors. On one hand, the setup of the mt-QSAR-LDA model provided information regarding structural requirements for higher ERK-1/2 inhibition. At the same time, it also helped to perform a fragment analysis, where the contributions of different molecular fragments for the inhibition of ERK-1/2 were estimated. The non-linear mt-QSAR-RF model, which was produced with an average accuracy of more than 96%, was used for screening of a focused kinase inhibitor library to retrieve the most potential virtual hits, which were then analysed from the aspects of fragment analysis. Finally, molecular docking and MD simulations were carried out with these drug-like virtual hits to estimate the binding energies of these virtual hits and also to understand their possible binding modes in ERK-1 and ERK-2 enzymes. The combination of the different in silico techniques employed in this work can provide important guidelines to facilitate the discovery of novel ERK-1/2 inhibitors.

## Figures and Tables

**Table 1:** Overall performance of the final multitarget quantitative structure–activity relationship (mt-QSTR) linear discriminant analysis (LDA) model.

Classification ^a^	Sub-Training Set	Test Set
ND_Total_^b^	3585	896
ND_active_^b^	1306	316
CCD_active_^c^	1256	310
Sensitivity(%)	96.17	98.10
ND_inactive_^b^	2279	580
CCD_inactive_^c^	1779	510
Specificity (%)	89.03	87.93
F-measure	0.893	0.891
Accuracy (%)	91.63	91.52
MCC	0.831	0.832

^a^ Classification parameters, ^b^ ND: Number of datapoints, ^c^ Correctly classified datapoints.

**Table 2:** Degree of collinearity among the variables of the mt-QSAR-LDA model.

Descriptors	*D[Tnsq5(CH)N2]_me_*	*D[Tnsq3(CH)MN]_bt_*	*D[Tssq11(CH)MN]_me_*	*D[Tssq5(POL)MX]_me_*	*D[Tnsq1(PSA)GM]_me_*	*D[Tnsq13(VDW)N2]_bt_*	*D[Tnsq2(HYD)N1]_bt_*
*D[Tnsq5(CH)N2]_me_*	1.000	−0.779	−0.613	0.225	0.218	−0.015	0.059
*D[Tnsq3(CH)MN]_bt_*	−0.779	1.000	0.606	−0.092	−0.277	0.093	0.073
*D[Tssq11(CH)MN]_me_*	−0.613	0.606	1.000	0.097	0.015	0.176	−0.130
*D[Tssq5(POL)MX]_me_*	0.225	−0.092	0.097	1.000	0.127	0.461	0.139
*D[Tnsq1(PSA)GM]_me_*	0.218	−0.277	0.015	0.127	1.000	0.158	−0.259
*D[Tnsq13(VDW)N2]_bt_*	−0.015	0.093	0.176	0.461	0.158	1.000	0.451
*D[Tnsq2(HYD)N1]_me_*	0.059	0.073	−0.130	0.139	−0.259	0.451	1.000

**Table 3:** Molecular descriptors of the mt-QSAR-LDA model and their respective definitions.

Descriptor	Description
*D[Tnsq13(VDW)N2]b_t_*	Total atom-based non-stochastic quadratic index of order 13 weighted by the van der Waals volume, modified by the Euclidean distance as mathematical operator, and depending on the chemical structure and the target
*D[Tnsq3(CH)MN]b_t_*	Total atom-based non-stochastic quadratic index of order 3 weighted by the charge, modified by the minimum value as mathematical operator, and depending on the chemical structure and the target
*D[Tssq5(POL)MX]m_e_*	Total atom-based stochastic quadratic index of order 5 weighted by the polarizability, modified by the maximum value as mathematical operator, and depending on the chemical structure and the measure of effect
*D[Tnsq2(HYD)N1]m_e_*	Total atom-based non-stochastic quadratic index of order 2 weighted by the hydrophobicity, modified by the Manhattan distance as mathematical operator, and depending on the chemical structure and the measure of effect
*D[Tnsq5(CH)N2]m_e_*	Total atom-based non-stochastic quadratic index of order 5 weighted by the charge, modified by the Euclidean distance as mathematical operator, and depending on the chemical structure and the measure of effect
*D[Tnsq1(PSA)GM]m_e_*	Total atom-based non-stochastic quadratic index of order 1 weighted by the polar surface area, modified by the geometric mean as mathematical operator, and depending on the chemical structure and the measure of effect
*D[Tssq11(CH)MN]m_e_*	Total atom-based stochastic quadratic index of order 11 weighted by the charge, modified by the minimum value as mathematical operator, and depending on the chemical structure and the measure of effect

**Table 4:** Overall performance of the final mt-QSTR-random forest (RF) model.

Classification ^a^	Sub-Training Set (10-Fold CV)	Test Set
ND_Total_^b^	3585	896
ND_active_^b^	1306	316
CCD_active_^c^	1239	304
Sensitivity(%)	94.87	96.20
ND_inactive_^b^	2279	580
CCD_inactive_^c^	2209	559
Specificity (%)	96.93	96.38
F-measure	0.962	0.948
Accuracy (%)	96.18	96.32
MCC	0.918	0.920

^a^ Classification parameters, ^b^ ND: Number of datapoints, ^c^ Correctly classified datapoints.

**Table 5:** Autodock binding energy values of the virtual hits (**H1**–**H19**) in ERK-1 and ERK-2 enzymes.

Cpd	Rigid Docking		Flexible Docking	
	ERK-1 (4QTB)	ERK-2 (4QTA)	ERK-1 (4QTB)	ERK-2 (4QTA)
**H1**	−9.48	−10.22	−10.79	−10.87
**H2**	−9.64	−9.25	−10.21	−10.39
**H3**	−9.36	−9.7	−10.27	−9.87
**H4**	−8.99	−8.96	−10.74	−9.72
**H5**	−9.68	−10.69	−9.84	−11.23
**H6**	−9.63	−9.35	−10.31	−10.8
**H7**	−8.92	−9.03	−10.48	−10.97
**H8**	−9.65	−10.52	−10.83	−9.78
**H9**	−9.28	−9.51	−10.19	−10.21
**H10**	−9.63	−10.19	−10.55	−9.62
**H11**	−9.46	−10.12	−10.06	−10.27
**H12**	−9.21	−9.56	−10.51	−9.47
**H13**	−9.21	−9.39	−10.3	−10.63
**H14**	−9.24	−10.06	−10.02	−9.59
**H15**	−9.19	−9.51	−9.62	−10.06
**H16**	−9.16	−9.75	−9.92	−9.93
**H17**	−9.63	−10.15	−10.43	−10.54
**H18**	−10.07	−9.9	−10.76	−10.58
**H19**	−9.16	−9.24	−10.86	−11.01
**Ulixertinib**	−8.77	−8.38	−9.97	−9.73

**Table 6:** Calculated binding free energies [ΔG_bind_] of the ERK-1/2 bound ligands.

Complexes	ΔG_Bind_
ERK2-H1	−33.46
ERK1-H1	−23.28
ERK2-ULX	−27.44
ERK1-ULX	−21.38

**Table 7:** Physicochemical properties of the virtual hits.

NAME	MW	nHDon	nHAcc	ALOGP
**H1**	457.56	2	8	4.18
**H2**	457.56	2	8	4.20
**H3**	427.48	1	9	2.16
**H4**	440.52	1	8	3.34
**H5**	442.49	2	9	3.15
**H6**	426.49	1	8	2.88
**H7**	426.49	1	8	3.31
**H8**	431.47	1	10	2.55
**H9**	429.50	1	8	3.11
**H10**	442.49	2	9	1.84
**H11**	441.51	1	9	2.53
**H12**	443.53	1	8	2.76
**H13**	429.50	1	8	3.11
**H14**	441.51	1	9	2.53
**H15**	443.53	1	8	2.76
**H16**	427.48	1	9	2.16
**H17**	426.49	1	8	2.88
**H18**	440.52	2	8	3.94
**H19**	441.51	2	9	2.79

## Supplementary Materials

The following are available online, SM1.xlsx, SM2.xlsx, SM3.xlsx and SM1.docx.
