# Supplementary material for: Multi-Target Chemometric Modelling, Fragment Analysis and Virtual Screening with ERK Inhibitors as Potential Anticancer Agents
Source: Molecules. 2019 Oct 30;24(21):3909. doi: 10.3390/molecules24213909 (PMC6864583; doi:10.3390/molecules24213909)
Supplement: Supplementary file 1 [file molecules-24-03909-s001.zip › SM1.pdf]

Supporting Information

Multi-Target Chemometric Modelling, Fragment Analysis and Virtual Screening with ERK Inhibitors as Potential Anticancer Agents

Amit Kumar Halder *, Amal Kanta Giri and Maria Natália Dias Soeiro Cordeiro *

Department of Chemistry and Biochemistry, University of Porto, 4169-007 Porto, Portugal

* Correspondence: amit.halder@fc.up.pt (A.K.H.); ncordeir@fc.up.pt (M.N.D.S.C.); Tel.:+351-22-040-2502 (M.N.D.S.C)

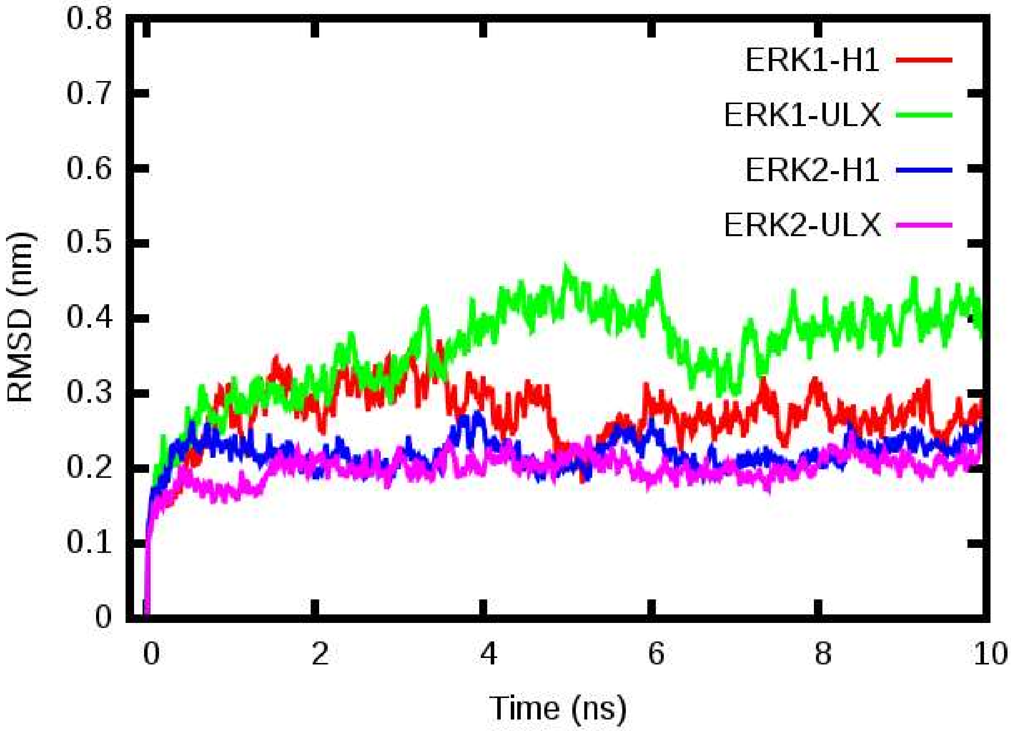

Figure S1. The root mean square deviation (RMSD) plots of backbone atoms of the receptor-ligand complexes.

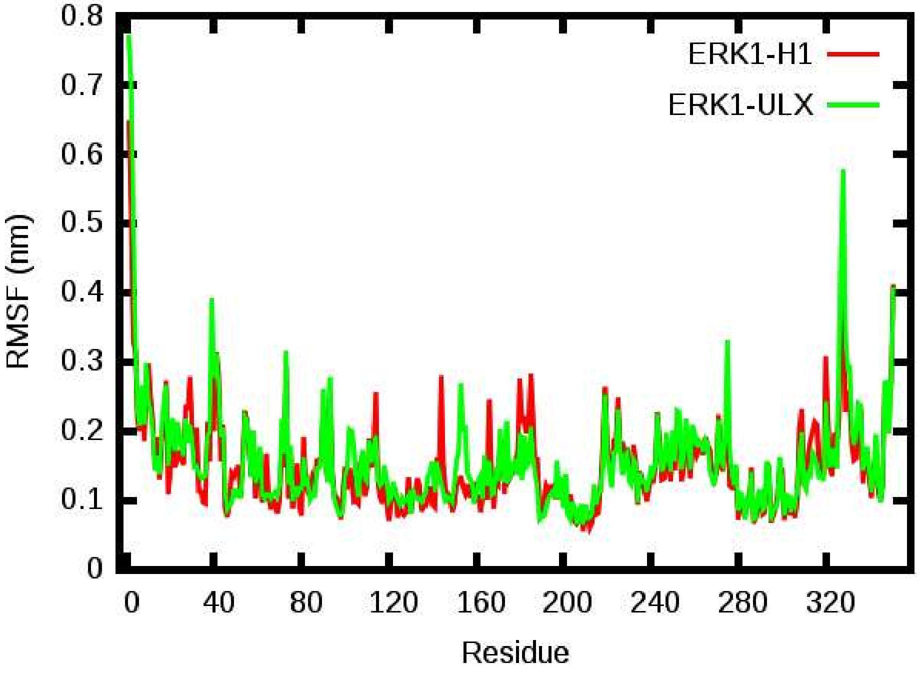

Figure S2. The root mean square fluctuation (RMSF) plots of ERK-1 complexes.

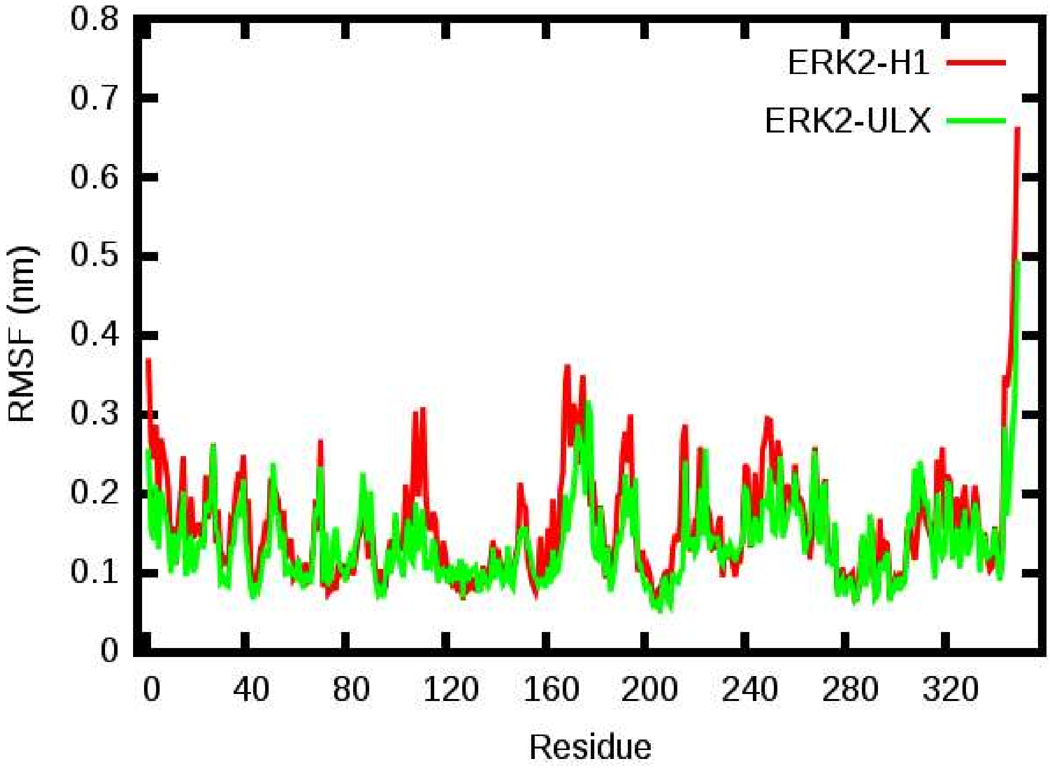

Figure S3. The root mean square fluctuation (RMSF) plots of ERK-2 complexes.

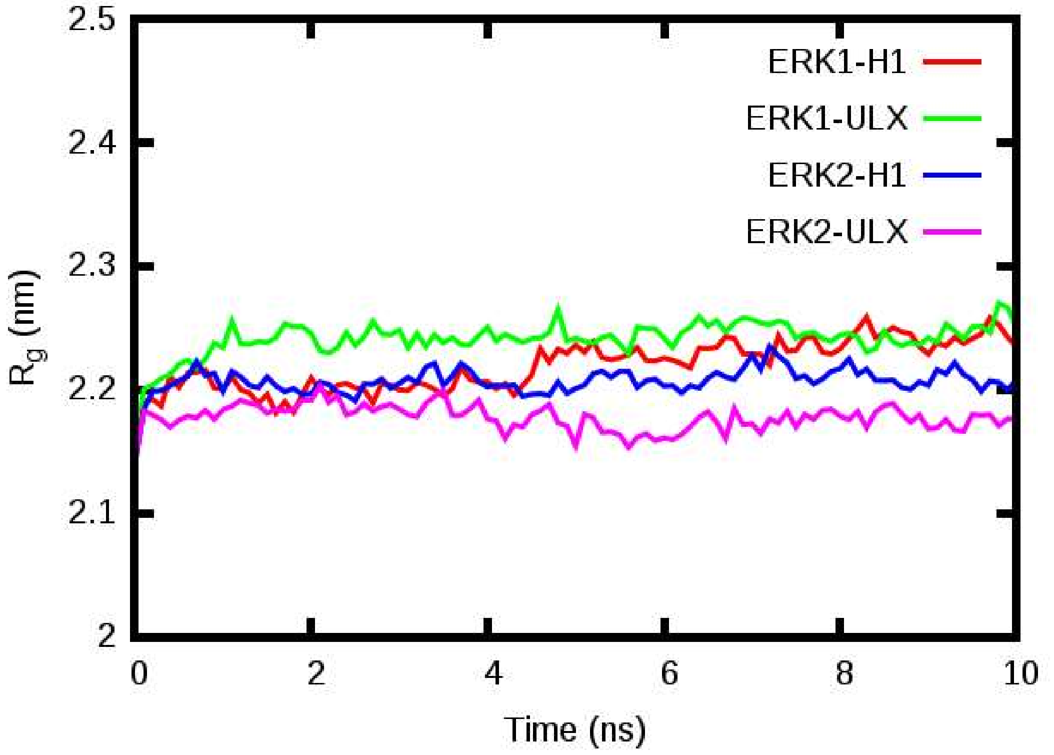

Figure S4. The radius of gyration (Rg) plots of ERK-1 and ERK-2 complexes

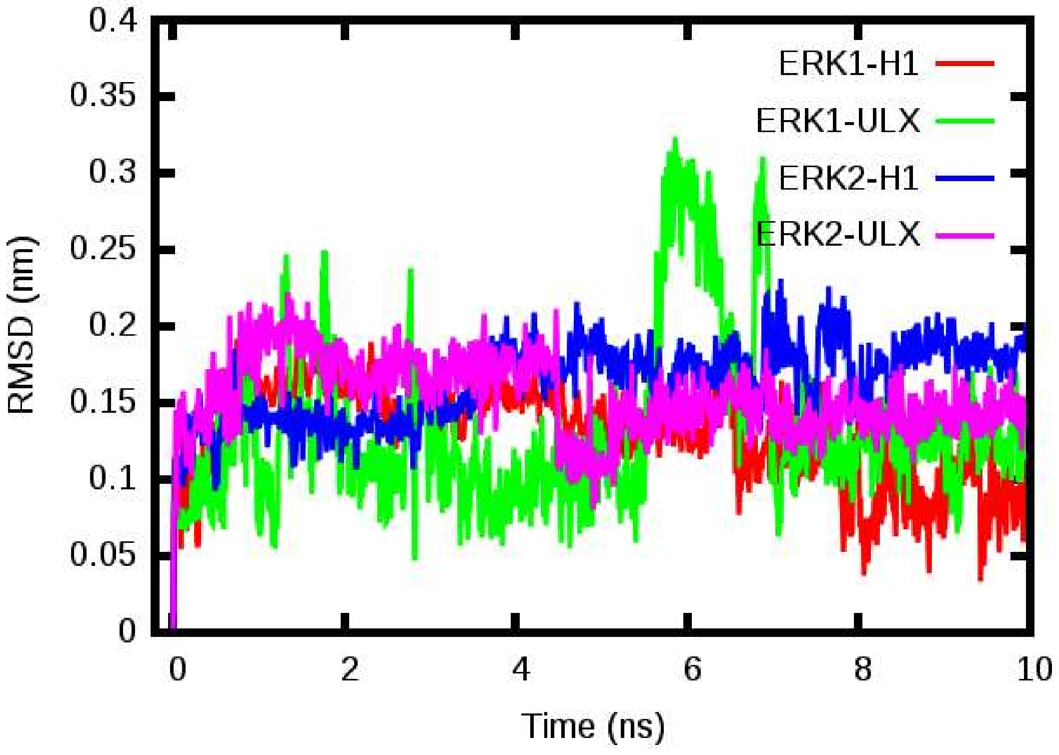

Figure S5. The root mean square deviation (RMSD) plots of bound ligands of the receptor-ligand complexes.

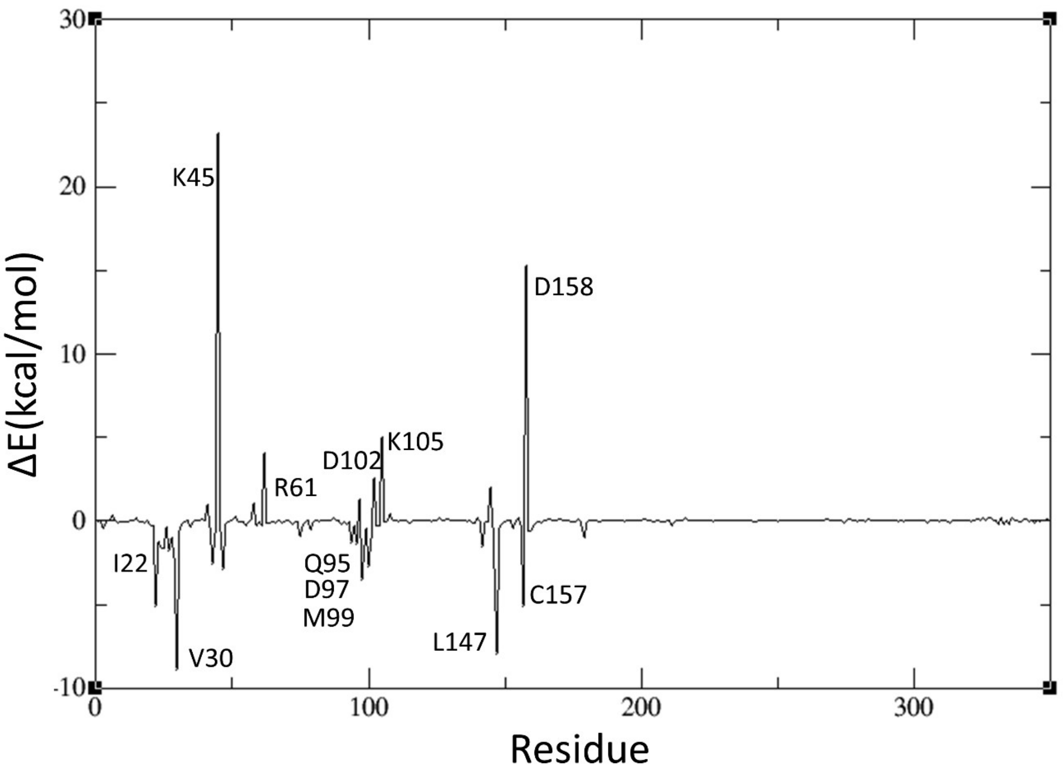

Figure S6. Per-residue decomposition profile of ERK2-ULX complex
